# Prevalence of intestinal helminths of red foxes (*Vulpes vulpes*) in central Europe (Poland): a significant zoonotic threat

**DOI:** 10.1186/s13071-018-3021-3

**Published:** 2018-07-28

**Authors:** Jacek Karamon, Joanna Dąbrowska, Maciej Kochanowski, Małgorzata Samorek-Pieróg, Jacek Sroka, Mirosław Różycki, Ewa Bilska-Zając, Jolanta Zdybel, Tomasz Cencek

**Affiliations:** National Veterinary Research Institute, al. Partyzantow 57, 24-100 Pulawy, Poland

**Keywords:** Red fox, Helminths, *Alaria alata*, *Echinococcus multilocularis*, Hookworms, *Mesocestoides*, *Taenia*, *Toxocara*, Poland

## Abstract

**Background:**

The red fox (*Vulpes vulpes*) is widely distributed in the world; in central Europe, it is the most numerous wild species of the family Canidae. It can play the role of a definitive host for many intestinal parasites, including zoonotic helminths. Poland, with its geographical location (central Europe), is an interesting area for parasitological investigations of this species. The aim of this study was to evaluate and compare the prevalence of intestinal helminths in red foxes in different regions of Poland.

**Methods:**

Intestines of 473 red foxes from four different regions were examined using the sedimentation and counting technique (SCT). In addition, 344 samples of faeces were examined using flotation.

**Results:**

Overall, intestinal helminths were found in 98.9% of red foxes. The average prevalence of detected parasites was as follows: *Mesocestoides* spp. (84.1%); hookworms (67.9%); *Alaria alata* (61.5%); *Toxocara/Toxascaris* (49.5%); *Taenia* spp. (42.5%); *Echinococcus multilocularis* (25.6%); and *Trichuris vulpis* (2.3%). The prevalence of the majority of parasite species was similar in each region. Significant differences between regions were observed in the case of *E. multilocularis*: a low prevalence in the south-western and northern regions (0% and 0.9%, respectively) and a high prevalence in the south-east and northeast (39.3% and 42.7%, respectively). In the case of *A. alata*, important differences were found between northern (96.5% and 93.7% in northern and northeast regions, respectively) and southern regions (15.2% and 24.7% for south-western and south-east regions, respectively). The percentage of positive samples obtained with coproscopic examination (except for *Trichuris*) was significantly lower than that obtained with SCT. Analysis of the prevalence estimated in individual regions with the use of both methods (flotation and SCT) showed a high correlation for all parasite species (except for *Mesocestoides* spp.). The flotation method also allowed us to detect the eggs of the lung nematode *Eucoleus aerophilus* (syn. *Capillaria aerophila*) (76.2% of positive foxes).

**Conclusions:**

This study showed a very high percentage of red foxes infected with intestinal helminths in different parts of Poland. Depending on the location, some differences were observed regarding the prevalence of dangerous zoonotic parasites, which should be considered in the assessment of infection risk for humans.

## Background

The red fox (*Vulpes vulpes*) is a widely distributed animal in the world. This species is spread throughout the northern hemisphere. The range of its occurrence increases with human expansion, through which it was even introduced into Australia (in the nineteenth century). In central Europe it is the most numerous wild species of the family Canidae. From the late 20th century to the first decade of the 21st century, a rapid increase (approximately four-fold) in the number of red foxes has been observed in Poland [1]. This increase was associated mainly with anti-rabies campaigns but also with the excellent adaptive abilities of this species to different environmental and nutritional conditions.

The red fox can act as a definitive host for many parasites, including zoonotic intestinal helminths, the most dangerous of which is *Echinococcus multilocularis*, a tapeworm whose larval forms cause alveolar echinococcosis in people. Mainly due to spreading of this infection in Europe in recent decades [2], a number of monitoring studies have been conducted to estimate the prevalence of *E. multilocularis* in red foxes, which sometimes also concerned other intestinal parasites [3–9]. These investigations showed that red foxes in Europe can also be the reservoir for other zoonotic parasites, such as *Toxocara canis*, or parasites that are potentially pathogenic for people, such as *Alaria alata*, hookworms and *Trichuris vulpis*.

Red foxes usually inhabit forests, fields and agricultural areas but are also perfectly adapted to living closely to people (in rural and urban areas). This means that the invasive eggs of zoonotic helminths dispersed in the environment are easily accessible to humans. Pets (dogs and cats), which are the final hosts of some of the red fox’s parasites (e.g. *E. multilocularis* and *T. canis*), can also be infected and become the next (closer) source of infection for humans [10–12].

In Poland, most of the parasitological studies in red foxes concerned only *E. multilocularis* [1, 13–18]. Only a few investigations also concerned other intestinal parasites, but they were conducted 15–25 years ago [19–21] or were in a very limited area, such as the Primeval Augustów Forest [22]. Poland, with its geographical location (central Europe) and as a place of impact of both the western and eastern historical migration routes of red foxes [23, 24], is an interesting area for such investigations. Therefore, the aim of this study was to evaluate and compare the prevalence of intestinal helminths in red foxes in different regions of Poland.

## Methods

### Samples

**Fig. 1:**
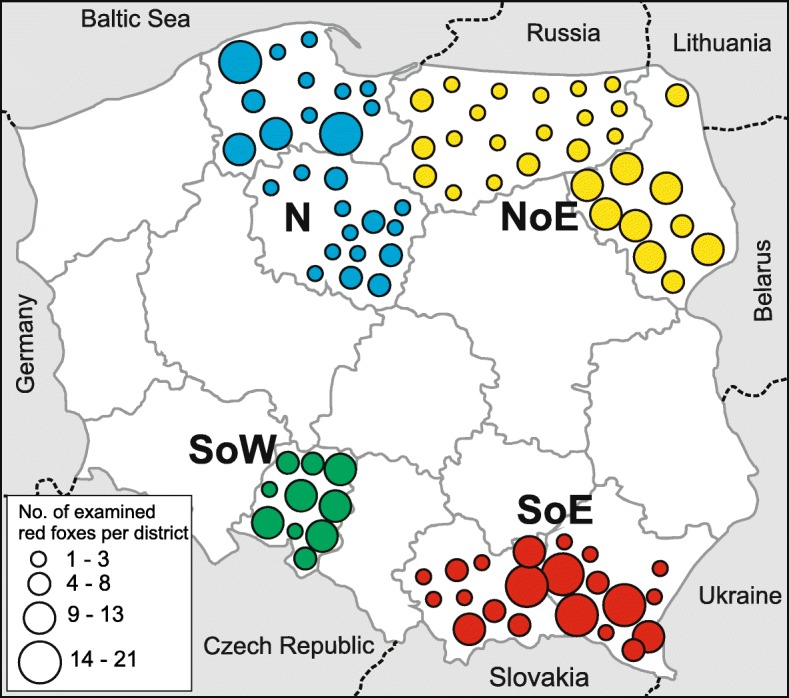
Distribution of the red foxes used in the investigation. Number of foxes obtained in individual districts (smaller administrative unit, parts of Polish provinces) were schematically presented by wheels in different sizes. Regions: N, North (blue); NoE, North-East (yellow); SoE, South-East (red); SoW, South-West (green)

Four hundred and seventy-three red foxes (*Vulpes vulpes*) shot (during an official survey concerning the efficacy of an anti-rabies vaccination) in different parts of Poland from 2011 to 2013 were used in the study. These samples were part of the material obtained previously in the context of an *E. multilocularis* monitoring study [13]. The investigation areas were divided into the following 4 regions: North (N) (*n* = 114) (Pomorskie and Kujawsko-Pomorskie Province), North-East (NoE) (*n* = 143) (Warmińsko-Mazurskie and Podlaskie Province), South-East (SoE) (*n* = 150) (Małopolskie and Podkarpackie Province) and South-West (SoW) (*n* = 66) (Opolskie Province). The study areas were selected to have a kind of cross-section of various landscape conditions in Poland. The northern regions are generally plains and large part of them included the Polish lakelands. However, the southern regions are characterized by smaller area of surface water, and in addition, the south-east region is typically mountainous area. Details concerning the distribution of sample collections, with the number of foxes per districts (smaller administrative unit, parts of Polish provinces) are presented in Fig. 1. The investigation materials were the intestines: the small and large intestines together (in the cases of 407 red foxes from North, South-East and North-East) and only the small intestines from 66 animals (SoW). The samples were frozen for at least 10 days at -80 °C before examination for safety reasons.

### Examination of intestines and faeces

The intestines were examined using the sedimentation and counting technique (SCT) [25, 26]. In addition, faecal samples (1–2 g) were collected from the rectums of 344 intestinal samples and were examined by flotation (the McMaster method according to Raynaud’s modification with the use of saturated magnesium sulfate, specific gravity 1.22 [27]) to detect parasite eggs and oocysts.

### Statistical analysis

Differences in the prevalence of the individual infections among regions were estimated by a chi-square test (or chi-square with Yates correction) with Bonferroni correction for multiple comparisons. Differences between the results obtained with SCT and flotation were calculated by a chi-square test (or chi-square with Yates correction). Pearson correlation factors were calculated for the results obtained in individual regions with the use of both methods (SCT and flotation). Arithmetic mean values and coefficients of variation were calculated for the intensity of infections and numbers of eggs or oocysts per gram of faeces. Confidence intervals of the percentages of infected foxes were calculated according to the method described by Newcombe [28]. The distribution of quantitative variables was tested by the Shapiro-Wilk test and the normality hypothesis of the data was rejected. Differences between multiple groups of the quantitative variables (intensity of infections) were determined by the Kruskal-Wallis test with Bonferroni correction. The differences in all analyses were considered statistically significant when *P* < 0.05. Statistical analyses were performed using Statistica 9.1 Stat Soft.

## Results

### Examination of intestines (sedimentation and counting technique)

In general, parasites were detected in 468 (98.9%) red foxes; only five foxes (one in N, SoW, NE and two in SoE) had no parasites in their intestines. Seven different types of parasites were found in the intestines: *E. multilocularis*, *A. alata*, *Mesocestoide*s spp., *Taenia* spp., *Toxocara/Toxascaris*, hookworms and *T. vulpis*. Overall, the most prevalent parasites were the tapeworms, *Mesocestoides* spp. (mean prevalence: 84.1%) followed by hookworms and *A. alata* (67.9% and 61.5%, respectively). The parasite with the lowest prevalence was *T. vulpis*; only approximately 2% foxes were positive. Detailed results are presented in Table 1.

**Table 1:** Prevalence of helminths in red foxes in different regions of Poland (estimated using SCT)

Species	Prevalence of parasites (%) (95% CI)				
	Overall (all regions) (*n* = 473)	South-East (*n* = 150)	North (*n* = 114)	North-East (*n* = 143)	South-West (*n* = 66)
*Alaria alata*	61.5 (57.1–65.8)	24.7^a^ (18.5–32.1)	96.5^b^ (91.3–98.6)	93.7^b^ (88.5–96.7)	15.2^a^ (8.4–25.7)
*Echinococcus multilocularis*	25.6 (21.9–29.7)	39.3^a^ (31.9–47.3)	0.9^b^ (0.2–4.8)	42.7^a^ (34.9–50.9)	0^b^ (0–5.5)
*Taenia* spp.	42.5 (38.1–47.0)	38.0 (30.6–46.0)	50.0 (41.0–59.0)	44.8 (36.9–52.9)	34.8 (24.5–46.9)
*Mesocestoides* spp.	84.1 (80.6–87.2)	92.0^a^ (86.5–95.4)	81.6^b^ (73.5–87.6)	81.1^b^ (73.9–86.7)	77.3^b^ (65.8–85.7)
*Toxocara*/*Toxascaris*	49.5 (45.0–54.0)	44.7 (37.0–52.7)	43.0 (34.3–52.2)	59.4 (51.3–67.1)	50.0 (38.3–61.7)
Hookworms	67.9 (63.5–71.9)	64.7 (56.7–71.9)	70.2 (61.2–77.8)	69.9 (62.0–76.8)	66.7 (54.7–76.9)
*Trichuris vulpis*	2.3 (1.3–4.1)	1.3 (0.4–4.7)	4.4 (1.9–9.9)	2.8 (1.1–7.0)	na
Overall (all parasites)	98.9 (97.6–99.6)	98.7 (95.3–99.6)	99.1 (95.2–99.8)	99.3 (96.1–99.9)	98.4 (91.6–99.6)

^a,b^Different letters in superscript indicate statistically significant differences in the prevalence of the parasites among regions (*P* < 0.05)

*Abbreviation:* *na* not applicable, *CI* confidence interval

**Fig. 2:**
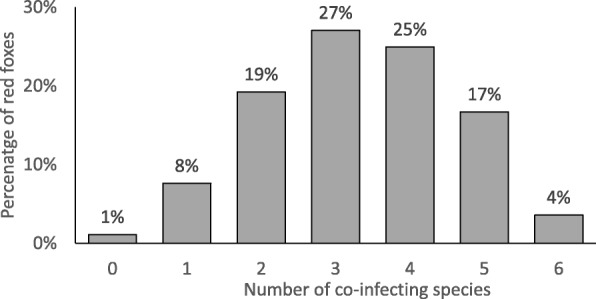
Distribution of the prevalence of co-infections. The total number of red foxes is 473

In most parasites (hookworms, *Taenia* spp., *Toxocara/Toxascaris* and *T. vulpis*), the prevalence of each species had similar levels in all regions. However, statistically significant differences were also observed. In the case of *E. multilocularis* differences were noted between North-East and North (*χ*^2^ = 40.51, *df* = 1, *P* < 0.0001), North-East and South-West (*χ*^2^ = 37.72, *df* = 1, *P* < 0.0001), South-East and North (*χ*^2^ = 52.38, *df* = 1, *P* < 0.0001), and South-East and South-West (*χ*^2^ = 33.76, *df* = 1, *P* < 0.0001). Significant differences in prevalence of *A. alata* were found between the northern and southern regions: between North and South-West (*χ*^2^ = 120.82, *df* = 1, *P* < 0.0001), North and South-East (*χ*^2^ = 132.51, *df* = 1, *P* < 0.0001), North-East and South-West (*χ*^2^ = 126.41, *df* = 1, *P* < 0.0001), North-East and South-East (*χ*^2^ = 140.77, *df* = 1, *P* < 0.0001). Moreover, significant differences in prevalence of *Mesocestoides* spp. were observed between South-East region and other regions: South-West (*χ*^2^ = 9.09, *df* = 1, *P* = 0.0026), North-East (*χ*^2^ = 7.51, *df* = 1, *P* = 0.0061), North (*χ*^2^ = 6.43, *df* = 1, *P* = 0.0070). Most red foxes (91.5%) were infected with more than one parasite species. Co-infections with three to four species were observed most frequently (27% and 25%, respectively) (Fig. 2).

Data for intensity of infection are presented in Table 2. An analysis of intensity showed significant differences between regions in the case of *A. alata* (*H* = 69.66, *df* = 3, *P* < 0.0001) and hookworm (*H* = 16.38, *df* = 3, *P* = 0.0009) infections. Multiple statistical comparisons of *A. alata* intensity showed significant differences between South-East and North (*P* < 0.0001), between South-East and North-East (*P* < 0.0001), between South-West and North-East (*P* = 0.0025), and between North and North-East (*P* = 0.0038). In the case of hookworms significant differences were detected between South-East and North (*P* = 0.0047) and between North-East and North (*P* = 0.0064).

**Table 2:** Intensity of helminth infections in red foxes in different regions of Poland estimated using SCT

Species		Overall (all regions) (*n* = 473)	South-East (*n* = 150)	North (*n* = 114)	North-East (*n* = 143)	South-West (*n* = 66)
*Alaria alata*	Mean	91.3	12.2^a^	94.4^b^	59.7^c^	15.1^a,b^
	Range	1–2540	1–110	1–2540	2–986	1–47
	CV (%)	205.4	177.9	268.1	250.5	94.3
*Echinococcus multilocularis*	Mean	4473.8	7215.0	9.0	1850.8	–
	Range	1–260,000	1–260,000	–	1–26,737	–
	CV (%)	584.9	511.4	–	280.8	–
*Taenia* spp.	Mean	10.3	11.7	7.1	11.2	12.3
	Range	1–200	1–200	1–42	1–98	1–56
	CV (%)	181.9	230.4	139.7	154.7	122.6
*Mesocestoides* spp.	Mean	112.9	138.2	87.4	103.7	110.0
	Range	1–2880	1–1500	1–1048	1–2880	1–840
	CV (%)	204.2	167.9	168.5	294.2	156.8
*Toxocara*/*Toxascaris*	Mean	5.0	5.2	3.2	5.5	5.8
	Range	1–40	1–40	1–22	1–35	1–28
	CV (%)	117.0	111.7	103.7	128.2	124.7
Hookworms	Mean	9.7	8.4^a^	14.3^b^	7.0^a^	10.2^a,b^
	Range	1–103	1–103	1–49	1–39	1–52
	CV (%)	145.0	185.2	124.9	125.5	112.3
*Trichuris vulpis*	Mean	1.2	1.5	1	1.3	na
	Range	1–2	1–2	–	1–2	na
	CV (%)	34.2	47.1	–	40.0	na

^a,b,c^Different letters in superscript indicate statistically significant differences in parasite intensity among regions (*P* < 0.05)

*Abbreviation:* *na* not applicable, *CV* coefficient of variation

### Examination of faeces (flotation)

The results of the coproscopic examination are presented in Table 3. *Capillaria* eggs were the most prevalent in all regions (mean prevalence of 76.2%). Because of the absence of *Capillaria* worms in the intestines, it should be classified as *Eucoleus aerophilus* (syn. *Capillaria aerophila*.), a nematode parasitising the lungs whose eggs are shed with the faeces. Eggs of trematodes and *Toxocara* sp. were found in a relatively high percentage of samples. Taeniid eggs (morphologically identical to *Taenia* spp. and *Echinococcus* spp.) were detected in 11.3% of foxes. The flotation method allowed us to detect coccidian infections; 7.6% of foxes shed coccidian oocysts (not identified to the species level).

**Table 3:** Results of faeces examination of red foxes in different regions of Poland

		Overall (all regions) (*n* = 344)	South-East (*n* = 140)	North (*n* = 92)	North-East (*n* = 112)
Trematoda	% Pos (95% CI)	43.3 (38.2–48.6)	7.9 (4.5–13.5)	62.0 (51.8–71.2)	72.3 (63.4–79.8)
	EPG (CV%)	917.4 (249.6)	37.3 (95.0)	754.0 (210.7)	1154.0 (241.8)
Taeniidae	% Pos (95% CI)	11.3 (8.4–15.1)	14.3 (9.5–21.0)	2.2 (0.6–7.6)	15.2 (9.7–23.0)
	EPG (CV%)	800.0 (298.9)	1244.3 (261.1)	115.0 (79.9)	331.8 (131.9)
*Mesocestoides* spp.	% Pos (95% CI)	3.5 (2.0–6.0)	2.1 (0.7–6.1)	6.5 (3.0–13.5)	2.7 (0.9–7.6)
	EPG (CV%)	668.8 (182.0)	1,400.0 (92.9)	599.0 (237.3)	69.0 (106.3)
*Toxocara* spp.	% Pos (95% CI)	25.0 (20.7–29.8)	20.0 (14.2–27.4)	22.8 (15.5–32.4)	33.0 (25.0–42.2)
	EPG (CV%)	321.9 (146.9)	344.4 (149.5)	341.9 (139.0)	289.4 (152.2)
*Toxascaris leonina*	% Pos (95% CI)	5.2 (3.3–8.1)	8.6 (5.0–14.4)	0.0 (0.0–4.0)	5.4 (2.5–11.2)
	EPG (CV%)	348.2 (109.0)	527.0 (109.3)	–	199.2 (76.6)
Hookworms	% Pos (95% CI)	15.4 (12.0–19.6)	10.0 (6.1–16.1)	30.4 (22.0–40.5)	9.8 (5.6–16.7)
	EPG (CV%)	276.6 (336.2)	577.6 (301.8)	209.5 (172.7)	64.1 (95.3)
*Trichuris vulpis*	% Pos (95% CI)	1.7 (0. 8–3.8)	0.7 (0.1–3.9)	4.3 (1.7–10.7)	0.9 (0.2–4.9)
	EPG (CV%)	128.1 (102.7)	88.3 (77.3)	90.0 (106.3)	400.0
*Capillaria* ^a^	% Pos (95% CI)	76.2 (71.4–80.4)	65.7 (57.5–73.1)	84.8 (76.1–90.7)	82.1 (74.0–88.1)
	EPG (CV%)	810.7 (230.6)	372.8 (186.0)	1,250.8 (231.4)	864.7 (164.2)
Coccidia	% Pos (95% CI)	7.6 (5.2–11.0)	10. 0 (6.1–16.1)	4.3 (1.7–10.7)	7.1 (3.7–13.5)
	OPG (CV%)	9785.6 (455.4)	17,283.6 (351.2)	271.5 (139.5)	1420.6 (172.7)

^a^Because of the lack of *Capillaria* worms in the intestines it should be classified as *Eucoleus aerophilus* (syn. *Capillaria aerophila*)

*Abbreviations:* *% Pos.* percentage of positive samples, *EPG* eggs per gram, *OPG* oocysts per gram, *CV* coefficient of variation, *CI* confidence interval

A comparison of results concerning the prevalence of parasites obtained with two different methods, SCT and flotation, are presented in Table 4. For this analysis only the samples from 344 foxes examined with both methods were used. Some data were adapted to allow comparisons prior to analysis. Thus, *E. multilocularis* and/or *Taenia* spp. results from SCT were combined in the Taeniidae group to compare with the positive taeniid flotation results (eggs of these two genera are morphologically identical) and separate results concerning *Toxocara* and/or *Toxascaris leonina* obtained in flotation were grouped with those of *Toxocara*/*Toxascaris* to compare with the same data obtained *via* SCT. In the case of each parasite (except for *Trichuris*) the percentage of positive results obtained with SCT was significantly higher than that obtained with the flotation method. Moreover, an analysis of prevalence estimated in individual regions using both methods (flotation and SCT) showed a high correlation for all parasite species (| *r* | values ranged between 0.857 to 0.984) except for *Mesocestoides* spp. (| *r* | = 0.175) (Table 4).

**Table 4:** Prevalence (%) of helminths in red foxes in different regions of Poland: comparison of SCT and flotation results

Region	Trematoda		Taeniidae		*Mesocestoides* spp.		*Toxocara*/ *Toxascaris*		Hookworms		*Trichuris vulpis*	
	SCT	Flot.	SCT	Flot.	SCT	Flot.	SCT	Flot.	SCT	Flot.	SCT	Flot.
Total (*n* = 344)	67.2^a^	43.3^a^	57.3^a^	11.3^a^	82.8^a^	3.5^a^	43.0^a^	29.9^a^	67.7^a^	15.4^a^	2.9	1.7
SoE (*n* = 140)	24.3^a^	7.9 ^a^	56.4^a^	14.3^a^	91.4^a^	2.1^a^	42.1^a^	28.6^a^	64.3^a^	10.0^a^	1.4	0.7
N (*n* = 92)	97.8^a^	62.0^a^	51.1^a^	2.2^a^	81.5^a^	6.5^a^	46.7^a^	22.8^a^	72.8^a^	30.4^a^	5.4	4.3
NoE (*n* = 112)	95.5^a^	72.3^a^	63.4^a^	15.2^a^	73.2^a^	2.7^a^	41.1^a^	37.5^a^	67.9^a^	9.8^a^	2.7	0.9
| r |^b^	0.984		0.857		0.175		0.888		0.903		0.962	

^a^There are significant differences between the prevalence estimated using SCT and flotation

^b^Absolute value of the correlation factor concerning the results obtained in individual regions with both methods (SCT and flotation)

*Abbreviations:* *SCT* sedimentation and counting technique, *Flot.* flotation method, *N* North region, *NoE* North-East region, *SoE* South-East region

## Discussion

The main diagnostic method used in this study was SCT; therefore, the results obtained by this technique were first used for the analysis of the real occurrence of parasites in red foxes. The investigation showed a very high percentage of infected red foxes; parasitic helminths were detected almost in all examined intestines (98.9%). Co-infections with three and four species occurred most frequently, similarly to the studies conducted in the Netherlands [3] or Poland [22].

The most prevalent parasites found in presented study were *Mesocestoides* spp. (mean prevalence of 84.1%); a very high prevalence was observed in all regions. These tapeworms were not identified to the species level. However, with reference to the molecular studies conducted by Zaleśny et al. [29] and Hrckova et al. [30], which showed that *Mesocestoides litteratus* was most commonly encountered species in Poland and Slovakia, it is probable that the majority of *Mesocestoides* tapeworms detected in our study may represent this species. A relatively high percentage of *Mesocestoides*-positive red foxes was noted previously in different regions of Poland (58–76%) [19–22]. A similarly high prevalence was observed in some other European countries, such as Lithuania (78%) [4] and northern Italy (82%) [31]. A wide distribution of *Mesocestoides* spp., mainly in a significant prevalence (27–68%), was confirmed by reports from many other European countries, i.e. Hungary [32], the Czech Republic [8], Denmark [5, 6], Slovenia [33] and eastern Germany [7, 34]. An absence or low prevalence of *Mesocestoides* spp. has been observed less frequently. For example, among more than 500 examined foxes, no *Mesocestoides* worms were detected in two independent studies in the UK, [35, 36]. In the Netherlands, only 5.7% positive foxes were found [3]. A low prevalence was noted also in northern Germany (Schleswig-Holstein) (1.9%) [37] and South Jutland (8.3%), the Danish region bordering Schlezwig-Holstein [38]. Especially interesting is the last result because in the same study, in other parts of Denmark (Copenhagen Province), as many as 78.6% of red foxes were infected [38]. Such large differences had no simple explanation; perhaps it was mainly associated with a lack of the specific intermediate host of the complex parasite life-cycle in this area.

The percentage of red foxes infected with hookworms did not differ significantly between the examined regions and was similar to the results obtained in Primeval Augustów Forest (located in north-eastern Poland) [22]. However, earlier studies conducted in western and southern Poland showed a significantly lower prevalence of these nematodes (11–35%) [19–21]. The difference may have resulted from the use of another diagnostic method (a modified intestinal scraping technique, IST). Hookworm species were not differentiated in our investigation, but the results of earlier studies suggest that most of them were *Uncinaria stenocephala* [20, 21]. This is also indicated by other studies conducted in Europe where *U. stenocephala* was found in a high percentage of red foxes (43–84%) [3, 4, 31, 33, 35, 36, 38, 39] alone or in a significant predominance over *Acylostoma caninum*, whose prevalence ranged between 0.6–13.2% [6, 38, 40].

The percentage of red foxes infected with *A. alata* varied significantly among the regions. In the northern regions, the prevalence was very high (above 90%), but several times lower in the southern areas (15.2% and 24.7% for south-western and south-east regions, respectively). A correlation could be seen between the prevalence of *A. alata* and the presence of surface water. A large part of the northern regions (North and North-East) is included in the Polish lakelands, but in the southern regions, the surface water area is much smaller. The presence of water reservoirs determines the occurrence of intermediate hosts (mainly planorbid snails) in the complex life-cycle of *A. alata*, which are associated with the aquatic environment. Similarly, a very high percentage of the red foxes infected with this fluke was found in north-eastern Poland (94%) [22], and a slightly lower percentage (57%) was found in central Poland [21]. Differences between the regions in western Poland were also observed previously, where in the north-western part of the country (rich in lakes), the prevalence was much higher (approximately 32%) than that in the south-western part (approximately 2%). A proportionally lower prevalence was obtained, probably because of the use of a different method (a modified IST). Outside Poland, a high percentage of *A. alata* infections (96%) was found in Lithuania, a country bordering Poland on the north-east [4]. Research in other European countries showed a moderately high or low prevalence of *A. alata* (5.5–31.4%) [3, 6, 7, 37, 38, 40, 41]. In some studies conducted in Europe, these trematodes were not found at all [8, 9, 35, 36, 39]. Our data showed that in regions with a high prevalence, the intensity of the infection was higher than that in regions with a low prevalence. A similar relationship was observed in Ireland, where the level of parasitism was highest in red foxes from those areas where the prevalence was the highest [41].

*Echinococcus multilocularis* is the most dangerous zoonotic parasite in temperate and cold climate zones. It occurs commonly in Europe, and its main definitive host is the red fox [2]. The monitoring of *E. multilocularis* in red foxes is very important because the prevalence of this parasite in this host species is an indicator of the epidemiological threat for humans. Our study revealed large differences in the prevalence of *E. multilocularis* among regions. A high prevalence was observed in the eastern regions (South-East and North-East), whereas in the North and South-West areas, only one infected fox was found. These results are part of the results obtained on a larger scale and discussed earlier in the publication concerning only *E. multilocularis* [13]. In these studies on *E. multilocularis* carried out throughout Poland, it was shown that the country is divided into two parts by the prevalence of this tapeworm: the western half with a low prevalence and the eastern half with a very high prevalence. The high prevalence in the eastern part of Poland corresponds to high percentage of infected foxes in countries bordering Poland from the east and south-east (Lithuania and Slovakia) [4, 42]. However, it is difficult to find the similar dependence concerning low prevalence in the western half - the available data from eastern Germany (bordering Poland from the west) indicated a relatively high percentage of infected foxes [43]. Moreover, other studies conducted in Poland indicated a gradual expansion of the zone with high prevalence towards the west [44]. Therefore, geographical differences in the occurrence of *E. multilocularis* in Poland are difficult to explain, these are probably the result of simultaneous influence of many factors (biological and physical) [13]. An attempt was made to explain these differences with an analysis of the genetic diversity of *E. multilocularis* in Poland [45, 46], but this has not yet given a definitive answer. The high prevalence of this species in the eastern regions represents a significant risk of infection for humans. In addition, cases of *E. multilocularis* in dogs [47] were also found in the area with a high prevalence (South-East), which further extended the potential for human infections.

*Toxocara* and/or *Toxascaris* spp. were found in a relatively high percentage of foxes (49.5%), and no differences in prevalence among regions were observed. Because of the similar morphology of the mature forms, nematodes of the genera *Toxocara* and *Toxascaris* were analysed together. However, the eggs of these two genera differ significantly, and the coproscopic examination showed that approximately 80% of the positive samples were *Toxocara canis* and the remaining were *Toxascaris leonina*. In other Polish studies, the prevalence of *T. canis* in red foxes was slightly lower (19.1–39.8%) [19–22]. In Europe, this nematode is commonly found in a medium or high percentage of red foxes (26.7–66.0%) [3–6, 9, 31, 33, 37–40, 48]. In most of the studies, only *T. canis* was identified. If *T. leonina* was also reported, it was usually (as in our study) with a much lower prevalence (0.6–18.1%) [3, 6, 33, 37, 48]. The exceptions are the studies from the Czech Republic, where the percentage of *T. leonina*-positive foxes was equal to that for *T. canis* [8], and from Slovakia, where *T. leonina* occurred more frequently [42]. The widespread occurrence of *T. canis* found in our study indicated a zoonotic threat, especially because the resistant eggs can accumulate in the environment (*T. canis* eggs can survive over winter in the soil and under a covering of snow even if the air temperature falls below -20 °C [49].

Tapeworms of the genus *Taenia* occurred in a relatively large percentage of foxes (42.5%) and the prevalence was similar in all examined regions. In other studies carried out in Poland, this proportion has been in the range between 22.2–40.9% [19–22]. In Europe *Taenia* spp. have been commonly registered in foxes with a prevalence ranging between 8.3–62% [3–7, 33–35, 37, 38, 40, 48]. It should be noted that the lowest percentage of infected foxes has been recorded in southern Europe (Italy; [40]), and the highest in Lithuania (the north-eastern part of the continent) [4]. Some authors identified *Taenia* tapeworms to the species level, the most frequiently identified species being *Taenia crassiceps*. Other species were also reported but with a low prevalence: *T. pisiformis*, *T. polyacanthus*, *T. hydatigena* and *T. taeniaeformis* [3, 33, 40, 48].

In this study, *T. vulpis* was characterised by the lowest prevalence (2.3%). This was supported by the results of other authors who reported between 0–4% of foxes infected with this nematode [6, 9, 19, 22, 33, 48]. Sometimes, this parasite was reported at a higher prevalence (16.1–16.3%) [3, 20]. However, *T. vulpis*, which is localized in the large intestine, was very often not taken into consideration during post-mortem parasitological investigations because surveillances in foxes were often limited to the small intestine (which is associated with monitoring focusing mainly on *E. multilocularis*).

The present study showed that evaluation of the prevalence with the use of coproscopy provided significantly poorer results compared to the real occurrence of the parasites in the intestines. The exception was the similar percentage of positive results obtained by SCT and flotation in the case of *T. vulpis*. In the present investigation, similar to our other study [22], the most significant difference between SCT and flotation was observed in the case of *Mesocestoides* infection, with more than 20-fold fewer positive samples in flotation than obtained by SCT. This is probably due to the specific egg structure of these tapeworms, which are difficult to isolate and identify in the preparation. The very low sensitivity of flotation in the detection of *Mesocestoides* infection was confirmed by Szell et al. [32]. These authors examined faeces from 180 foxes infected with *Mesocestoides* spp. and found no eggs of this tapeworm in any of the samples. The results of these authors, as well as the present data, suggest that with such a high prevalence of these tapeworms in foxes, it can also be high in dogs, but remains undetected in veterinary practices due to the limitations of the flotation method.

Although coproscopy is a less sensitive method, it provided the opportunity to see the differences in prevalence among regions. In the present investigation, in the majority of cases, there was a correlation between the percentage of positive results obtained with the use of SCT and flotation.

In addition, the microscopic examination of faeces made it possible to detect the infection of lung worms, *E. aerophilus* (syn. *C. aerophila*). Identification on the basis of egg morphology is difficult, but the absence of nematodes of *Capillaria* spp. as well as *Trichuris* (eggs with a similar morphology) in these intestines, confirmed that these were *E. aerophilus* eggs. The high percentage (76.2%) of positive results is very similar to the results obtained in Poland in the Augustów Primeval Forest [22]. Similar or higher prevalences of this parasite in foxes were found (during respiratory tract examination) in Denmark (80% [6]), Serbia (84% [50]), Norway (88% [51]) and Lithuania (93% [4]). Lower prevalences of this parasite were found in Hungary [52] and northern Italy [53] (61.7% and 41.8%), respectively. It is highly probable that the real prevalence of *C. aerophila* in Polish red foxes was higher since our results were based only on eggs detected in faeces. This may be supported by Lalosevic et al. [50] where 84% of foxes had *E. aerophilus* worms in their lungs but only 38% were positive in coproscopy.

## Conclusions

This study has shown a wide range of parasitic helminth infections in red foxes in Poland. For some parasites significant differences among the regions were observed, which were probably associated with environmental conditions. Dangerous zoonotic parasites (*E. multilocularis* and *T. canis*) were detected. Therefore, the impact of these parasites on human health should be taken into consideration in risk assessments.

## Abbreviations

EPG: Eggs per gram
IST: Intestinal scraping technique
N: North region
NoE: North-East region
OPG: Oocysts per gram
SCT: Sedimentation and counting technique
SoE: South-East region
SoW: South-West region
